# Fighting Sarcopenia in Ageing European Adults: The Importance of the Amount and Source of Dietary Proteins

**DOI:** 10.3390/nu12123601

**Published:** 2020-11-24

**Authors:** Diego Montiel-Rojas, Andreas Nilsson, Aurelia Santoro, Alberto Bazzocchi, Lisette C. P. G. M. de Groot, Edith J. M. Feskens, Agnes A. M. Berendsen, Dawid Madej, Joanna Kaluza, Barbara Pietruszka, Amy Jennings, Susan Fairweather-Tait, Giuseppe Battista, Miriam Capri, Claudio Franceschi, Fawzi Kadi

**Affiliations:** 1 School of Health Sciences, Örebro University, 702 81 Örebro, Sweden; diego.montiel@oru.se (D.M.-R.); fawzi.kadi@oru.se (F.K.); 2 Department of Experimental, Diagnostic and Specialty Medicine, Alma Mater Studiorum, University of Bologna, 40138 Bologna, Italy; aurelia.santoro@unibo.it (A.S.); g.battista@unibo.it (G.B.); miriam.capri@unibo.it (M.C.); claudio.franceschi@unibo.it (C.F.); 3 Alma Mater Research Institute on Global Challenges and Climate Change (Alma Climate), University of Bologna, 40126 Bologna, Italy; 4 Diagnostic and Interventional Radiology, IRCCS Istituto Ortopedico Rizzoli, 40136 Bologna, Italy; alberto.bazzocchi@ior.it; 5 Department of Human Nutrition and Health, Wageningen University, 6708WE Wageningen, The Netherlands; lisette.degroot@wur.nl (L.C.P.G.M.d.G.); edith.feskens@wur.nl (E.J.M.F.); agnes.berendsen@wur.nl (A.A.M.B.); 6 Department of Human Nutrition, Warsaw University of Life Sciences-SGGW, 02-776 Warsaw, Poland; dawid_madej@sggw.edu.pl (D.M.); joanna_kaluza@sggw.edu.pl (J.K.); barbara_pietruszka@sggw.edu.pl (B.P.); 7 Norwich Medical School, University of East Anglia, Norwich NR4 7TJ, UK; Amy.Jennings@uea.ac.uk (A.J.); S.Fairweather-Tait@uea.ac.uk (S.F.-T.); 8 Department of Applied Mathematics, Institute of Information Technology, Mathematics and Mechanics (ITMM), Lobachevsky State University of Nizhny Novgorod-National Research University (UNN), 603950 Nizhny Novgorod, Russia

**Keywords:** elderly, plant protein, animal protein, muscle mass, macronutrients, isocaloric substitution, muscle strength, physical activity, metabolic syndrome

## Abstract

While an adequate protein intake is important for the maintenance of muscle mass during ageing, the amount and source of protein necessary for optimal prevention of sarcopenia remains to be determined. The present study aimed to investigate the influence of the amount and source of dietary proteins on sarcopenia risk in a cohort of 65–79-year-old European adults within the frame of the NU-AGE study. A total of 986 participants were included in the analysis. Skeletal muscle index (SMI), assessed by dual-energy X-ray absorptiometry (DXA), and handgrip strength (HG) were employed to create a continuous sex-specific sarcopenia risk score (SRS). Total amount together with animal- and plant-derived sources of proteins were obtained from a 7-day food record. Differences in SRS were analysed across groups of total protein intake (<0.8 g/body weight (BW); 0.8–<1.0 g/BW; 1.0–<1.2 g/BW; and ≥1.2 g/BW). The association between SRS and the different sources of protein was assessed using isocaloric substitution models adjusted by demographic, medical, and lifestyle factors. A significant linear dose-response relationship was observed, with a lower SRS linked to higher protein intakes. Based on the isocaloric substitution modelling, a reduced SRS was observed when increasing plant protein to the detriment of animal protein, while holding total protein intake constant. Further, this result remained significant after stratifying the analysis by adherence to different levels of protein intake. Our findings suggest that older adults may benefit from increasing protein intakes above current recommendations. Besides total amount, protein source should be considered when promoting health dietary habits in older adults for the prevention of sarcopenia.

## 1. Introduction

The decline in muscle mass, strength, and related functional abilities accompanying the ageing process have received considerable attention due to its association with an array of negative health-related outcomes, such as falls, disability, loss of independence, and mortality [1]. In this respect, sarcopenia is defined as a progressive skeletal muscle disorder operationalised by low levels of muscle strength and muscle quantity, and with physical performance as an indicator of severity [2]. The progression of sarcopenia appears to be related to a complex interweaving of biological and environmental determinants as people age. Among others, lifestyle behaviours including physical activity and healthy dietary habits are considered key factors in counteracting age-related increases in sarcopenia risk [3,4,5]. In particular, adequate protein intake is recognised as a core dietary element for healthy ageing, where a large body of research highlights its links to muscle mass and function [6,7,8,9,10,11,12,13,14,15].

While the optimal amount of protein for maintenance of muscle health remains elusive, dietary allowances between 0.8 and 1.2 g/BW have been recommended for older adults [15,16,17,18,19,20,21,22,23]. Importantly, the shape of the dose-response relationship between total protein intake and indices of sarcopenia risk (i.e., muscle mass, strength) is yet to be determined and would increase our knowledge whether a potential ceiling effect exists above which no further beneficial impacts on sarcopenia risk are obtained. 

Besides total protein intake, it has been shown that animal-based proteins induce a higher stimulation of muscle protein synthesis [24,25], and higher physical function [10,26] compared to plant-based proteins in older people. Paradoxically, evidence also suggest a positive link between plant-based protein and muscle mass, strength, and functional performance [14,27,28,29,30,31]. Currently, how different sources of protein may provide the best benefit in sarcopenia prevention remains unclear and warrants further investigations in order to refine guidelines on healthy eating behaviours in general, and protein sources in particular, for promoting muscle health during aging. For this purpose, other factors of importance for preservation of muscle health, such as physical activity habits and metabolic health status need to be considered when depicting the role of proteins, both in term of total intake and food source. 

Taken together, as both quantity and quality of protein intake may have separated potential benefits on muscle health, it could be hypothesised that benefits linked to protein source may be accentuated by a given level of total protein intake. Therefore, integrating both quantitative and qualitative aspects when exploring links between proteins and muscle health would hold important clinical implications related to sarcopenia prevention in aging populations.

Therefore, the present study aimed to investigate whether different food sources of dietary proteins influence on sarcopenia risk across different levels of total protein intake in a cohort of older European adults.

## 2. Materials and Methods 

### 2.1. Participants

A total of 986 older men and women aged 65–79 years from four different countries (Italy, Poland, The Netherlands, and The United Kingdom) were recruited within the frame of the NU-AGE project (Clinicaltrials.gov, NCT01754012). A detailed description of the study design has been previously defined [32,33]. Written informed consent was obtained from each participant before starting and the whole study conducted in accordance with the Declaration of Helsinki. Local ethical approval was provided by independent committees in each recruiting centre.

### 2.2. Dietary Intake

Dietary intake was monitored by 1-week food record as previously described [32,34]. Briefly, participants followed a training in advance and received written instructions about how to complete a pre-formatted template which included 8 meal occasions during each day. Nutritional information were obtained by software exploiting local food references [32,34,35] (INRAN and IEO in Italy, WISP in the UK, NEVO 2011 in The Netherlands and NFNI in Poland). Total animal- and plant-based protein intakes were normalised to body weight (g/BW). Based on established protein intake recommendations [21,36], four groups were created according to the following intakes: <0.8 g/BW; 0.8–<1.0 g/BW; 1.0–<1.2 g/BW; and ≥1.2 g/BW.

### 2.3. Anthropometry and Body Composition

Height (cm) and body weight (kg) were obtained using a calibrated stadiometer and scale to the nearest 0.1 cm and kg, respectively. A whole-body dual-energy X-ray absorptiometry (DXA) scan was performed to determine total and regional body composition, including appendicular lean mass (kg). All the scanners were daily calibrated against a standard calibration block and DXA performed by trained technicians according to manufacturer instructions. 

### 2.4. Sarcopenia Risk Score

SMI was calculated based on appendicular lean mass as described elsewhere [37,38,39]. Handgrip strength was assessed with a Jamar handheld dynamometer (Patterson Medical, Warrenville, IL, USA) and normalised by body weight. The continuous clustered sarcopenia risk score (SRS) consisted in a sex-specific composite z-score, including skeletal muscle mass index (SMI) and handgrip strength, as previously described [40]. These variables are representative of muscle quantity and strength according to the most recent operational definition of sarcopenia by the European Work Group on Sarcopenia in Older People [2]. 

### 2.5. Adherence to Physical Activity Guidelines

Time spent in moderate-to-vigorous physical activity (MVPA) was monitored during a 7-day period using a waist-mounted Actigraph accelerometer (GT3x activity monitor, Actigraph, Pensacola, FL, USA) as previously described [41]. Participants spending 150 or more minutes per week in MVPA, based on the standardised cut-point of ≥2020 count per minutes [42], were classified as adhering to guidelines about health-enhancing physical activity (PA) [43]. 

### 2.6. Assessment of Metabolic Risk

Participants were categorised as having high or low metabolic risk according to the International Diabetes Federation (IDF) definition of metabolic syndrome (MetS) [44], as described elsewhere [45]. Standing waist circumference (WC), midway between the lowest rib and the iliac crest, was measured to the nearest 0.1 cm. Automated electronic blood pressure monitors were employed to assess systolic and diastolic blood pressures [39]. Blood glucose, high-density lipoprotein (HDL), and triglycerides levels were measured using standard methodologies.

### 2.7. Statistical Analysis

Data are presented as arithmetic means and standard deviations, unless indicated. Differences between groups of protein intake were determined by either one-way ANOVA with Sidak correction or chi-squared test with Bonferroni correction. Prior to analysis of links between protein intake and SRS, no interactions with either PA or MetS were observed, therefore, final analyses were based on the whole sample. Additionally, regression analysis was employed to assess the effect on SRS by isocaloric substitution of animal-based protein with plant-based protein [40,46]. Estimates are interpreted as changes in SRS with 0.1 g/BW increase of plant protein to the detriment of animal protein, while keeping total protein intake constant. The regression model included total protein intake, plant protein intake, and total energy intake and was adjusted. All models were adjusted for age, recruiting centre, adherence to PA guidelines, prevalence of MetS, medication, smoking habits, total energy intake, and fibre intake. All analyses were performed both on the whole population and after stratification by groups of protein intake. Assumptions behind parametric analyses, including normality, linearity, homogeneity of variance, and multicollinearity were checked. Small-to-moderate effect sizes on SRS were detectable with a power of >80% given our sample size and alpha set to 0.05. The analyses were conducted using SPSS version 27.

## 3. Results

A total of 417 men (aged 71.1 ± 4.1 years) and 569 women (aged 71.0 ± 3.9 years) were included in the final analysis. SMI and handgrip were 30.1 ± 3.2% and 0.49 ± 0.09 kg/BW for men, and 24.4 ± 2.8% and 0.37 ± 0.09 kg/BW for women, respectively. Basic characteristics presented across protein intake groups are shown in Table 1. Gender distribution (58% female) was equal across groups of protein intake. Notably, those belonging to the highest protein intake group (≥1.2 g/BW) had lower prevalence of MetS and medication use, as well as higher adherence to PA guidelines compared to those with protein intakes below 1.0 g/BW (Table 1).

Total energy intake and macronutrient distribution (E%) across protein intake groups are shown in Table 2. Importantly, an average of 63% of the total protein intake was derived from animal sources, with no significant differences between protein intake groups. 

**Figure 1:**
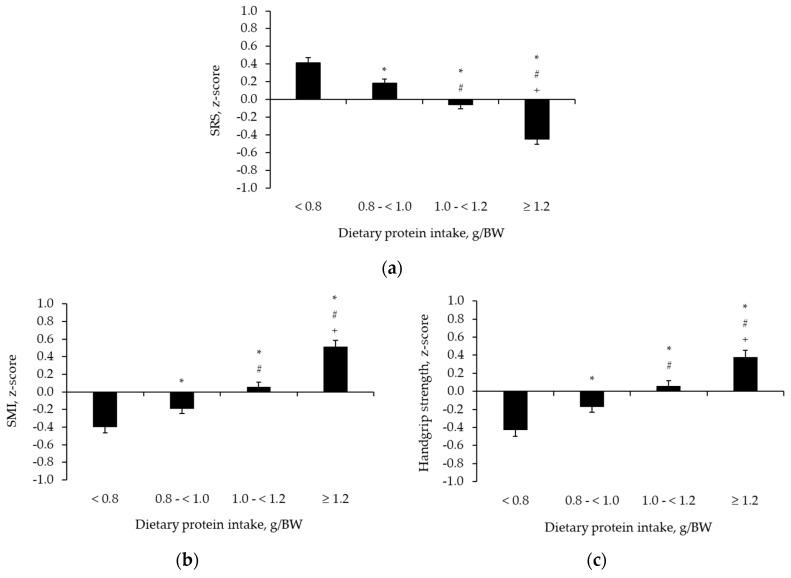
Differences in sarcopenia risk score (**a**), skeletal muscle index (**b**), and handgrip strength (**c**) across groups of protein intake. Data are estimated marginal means ± SEM adjusted for age, recruiting centre, medication, smoking habits, prevalence of MetS, adherence to PA guidelines, fibre intake, and total energy intake. * *p* < 0.05 vs. < 0.8 g/BW protein intake; ^#^ *p* < 0.05 vs. 0.8–<1.0 g/BW protein intake; and ^+^ *p* < 0.05 vs. 1.0–<1.2 g/BW protein intake.

Factorial ANOVA indicated a significant main effect of protein intake on SRS (*p* < 0.05), which remained after adjustment by covariates, including adherence to PA guidelines and prevalence of MetS. Importantly, post hoc-analysis revealed a linear dose-response manner of the main effect, where a shift in protein intake group was linked to a significantly lower SRS (*p* < 0.05) (Figure 1a). In addition, we extended the analysis to include main effects of protein intake groups on single components of SRS. The analysis revealed similar trends of the dose-response relationship between groups of protein intake and SMI and handgrip strength (Figure 1b,c).

The present study further investigated the potential role of protein sources on SRS using an isocaloric substitution model. Our analysis showed that replacing an equal amount of animal-derived proteins by plant-derived proteins was associated with a significantly reduced SRS (Table 3). Strikingly, the beneficial association on SRS by favouring plant-derived proteins in detriment of animal-derived proteins was evident across all protein intake groups (Table 3), thus indicating the role of protein source as independent of protein quantity.

## 4. Discussion

The present study addressed the question of whether protein source matters for sarcopenia risk in older adults when considering total protein intake and health-enhancing PA levels. Here we show that increasing the proportion of plant-derived to the detriment of animal-derived proteins is beneficially linked to lower sarcopenia risk in a cross-cultural sample of older European adults. Importantly, the favourable impact of plant-derived proteins was evident across a broad range of total protein intakes and independent of adherence to PA guidelines.

Our study suggests a beneficial role of increasing the relative amount of plant-derived to the detriment of animal-derived proteins in the prevention of sarcopenia. It has previously been shown that plant-derived proteins may represent an inadequate source of the essential amino acids lysine and leucine [47,48,49], with a reduced capability to stimulate muscle protein synthesis [50,51,52,53,54,55], thereby promoting animal-derived proteins as the primary source for maintenance of muscle mass and function in older adults. However, several studies examining effects of plant-derived proteins on protein synthesis were based on isolated and generally low digestible plant sources, such as soya and wheat [24,25,51,52,56]. Interestingly, it has been suggested that no deficits regarding essential amino acids such as lysine would be expected to occur with relative intakes of plant-derived proteins below 70% of total intake [57]. Therefore, given an average consumption of 37% plant-derived proteins in our study population, which is in line with previous data [58,59], alteration of the protein source distribution in favour of plant-derived proteins would be feasible with safe margins against any deficiencies in composition of essential amino acids.

The notion that animal-derived proteins are of particular importance for muscle health has been challenged by recent reports showing that greater intakes of plant-derived proteins are related to faster walking speed [14,27], and higher scores on the short physical performance battery using copula graphical models [29]. Further, plant-derived proteins have also been associated to reduced loss of muscle over a 4-year period in older adults [28]. To date, the beneficial impacts of plant-derived proteins on indices of muscle health are not fully explained. It has been suggested that alkaline plant-based diets, rich in minerals such as potassium and magnesium, may favour maintenance of muscle mass and function in older adults [60]. Further research is warranted in order to unravel links between plant-derived proteins and muscle health.

Interestingly, the role of protein source in prevention of age-related muscle wasting might be partly influenced by the total protein intake. For example, previous reports indicated that an adequate amount of protein intake would attenuate any benefits related to protein sources [30,55,61,62]. In this respect, an important finding in this study was that beneficial links of plant-derived proteins with SRS and its components were evident across groups of protein intakes ranging from below 0.8 g/BW to above 1.2 g/BW. Thus, impacts of protein source on muscle health seem independent of protein consumption over a broad range of intakes in this population of older European men and women. 

Our data not only confirm previously established links between total protein intake and muscle mass and function [6,8,9,10,11,15,63,64], it further highlights a linear dose-response pattern indicating no apparent ceiling effect within protein intakes ranging from 0.8 to 1.2 g/BW. Indeed, it is suggested that maximal stimulation of muscle protein synthesis requires a greater relative protein intake in older compared to younger adults [65]. Moreover, our analysis encompassed several other factors with potential to influence on sarcopenia risk, including physical activity habits. Taken together, our data extend current guidelines on diets for healthy aging, advocating greater protein intakes (1.2 g/BW) to older adults regardless of adherence to guidelines on health-enhancing physical activity.

A strength of the present study is the inclusion of older adults from four European countries, which allows to capture the role of dietary proteins on sarcopenia risk in geographically and culturally diverse European populations. Moreover, the analysis encompassed several potential confounding factors, including demographic, biologic. and behavioural aspects. The use of food record for the estimation of protein intakes, DXA-derived assessment of muscle mass, and objective assessment of physical activity in a large cohort of older adults further strengthen the validity of our analyses. However, this study is not without limitations. The cross-sectionally-based analysis of isocaloric substitution of plant- and animal-derived proteins precludes conclusions about causality, and even though several covariates were considered we cannot rule out presence of residual confounding. Moreover, caution should be taken when extending the study findings to populations including older adults with frailty and overt disease. 

## 5. Conclusions

The present study highlights the importance of both, amount and source of proteins for sarcopenia risk in older adults. The linear dose-response pattern between protein amount and sarcopenia risk, supports the promotion of protein intakes even above 1.2 g/BW in aging populations. Further, modifying distribution of protein source in favour of plant-derived proteins is beneficially linked to sarcopenia risk across a broad range of total protein intakes. This emphasizes the dual roles of protein quantity and quality in preservation of muscle mass and function in older adults, regardless of adherence to guidelines on health-enhancing physical activity. 

## Figures and Tables

**Table 1:** General characteristics of the study population by groups of protein intake.

	Protein Intake ^a^				
	<0.8	0.8–<1.0	1.0–<1.2	≥1.2	
*n*	205	296	279	206	
**Basic Characteristics**					
Age, y	71.7 ± 4.0	71.1 ± 4.1	70.9 ± 3.8	70.4 ± 3.9 *	
Weight, kg	83.3 ± 13.8	75.6 ± 11.9 *	70.9 ± 11.7 * ^#^	66.9 ± 11.1 * ^# +^	
Height, cm	166.7 ± 9.4	165.9 ± 8.8	164.5 ± 9.5	165 ± 9.2	
Smoking, % never	46.1	45.9	55.6	58.3 *	
Medication, % yes	85.3	82.2	70.6 * ^#^	71.8 * ^#^	
PA. guidelines, % yes	44.1	51.0	55.2	67 * ^#^	
MetS, % yes	63.7	45.6 *	32.3 * ^#^	27.2 * ^#^	

Continuous data are expressed as mean ± SD or are otherwise indicated. ^a^ g/BW. BW: Body weight; PA: Physical activity; and MetS: Metabolic syndrome. * *p* < 0.05 vs. <0.8 g/BW protein intake; ^#^ *p* < 0.05 vs. 0.8–<1.0 g/BW protein intake; and ^+^ *p* < 0.05 vs. 1.0–<1.2 g/BW protein intake.

**Table 2:** Macronutrient and energy intake of the study population by protein intake groups.

	Protein Intake ^a^			
	<0.8	0.8–<1.0	1.0–<1.2	≥1.2
*n*	205	296	279	206
Total energy intake, Kcal	1510 ± 327	1718 ± 310 *	1878 ± 373 * ^#^	2143 ± 440 * ^# +^
Carbohydrates, E%	49.5 ± 7.1	49.3 ± 6.6	48.5 ± 6.2	47.9 ± 7.4
Fat, E%	31.1 ± 5.2	30.9 ± 5.4	31.2 ± 5.3	31.4 ± 6.0
Protein, E%	15.7 ± 2.6	16.3 ± 2.4	17.0 ± 2.6 * ^#^	17.5 ± 2.7 * ^#^
Plant Protein, % total protein	37.4 ± 9.9	36.5 ± 8.8	36.7 ± 9.6	36.7 ± 9.6
Animal Protein, % total protein	62.6 ± 9.9	63.5 ± 8.8	63.3 ± 9.6	63.3 ± 9.6

Continuous data are expressed as mean ± SD. ^a^ g/BW. E%: Percentage of total energy. * *p* < 0.05 vs. <0.8 g/BW protein intake; ^#^ *p* < 0.05 vs. 0.8–<1.0 g/BW protein intake; and ^+^ *p* < 0.05 vs. 1.0–<1.2 g/BW protein intake.

**Table 3:** Isocaloric substitution of animal-based protein with plant-based protein on sarcopenia risk.

	Sarcopenia Risk Score		
Model	β-Coeff.	95% CI	*p*-Value
**Whole Population**			
Plant Protein	−0.249	−0.303 to −0.196	<0.001
**<0.8 g/BW**			
Plant Protein	−0.323	−0.498 to −0.149	<0.001
**≥0.8–<1.0 g/BW**			
Plant Protein	−0.198	−0.318 to −0.078	0.001
**≥1.0–<1.2 g/BW**			
Plant Protein	−0.276	−0.364 to −0.189	<0.001
**≥1.2 g/BW**			
Plant Protein	−0.234	−0.335 to −0.133	<0.001

CI: Confidence interval. Substitution model contains total protein intake, plant protein intake, and total energy intake. Models were additionally adjusted for age, recruiting centre, medication, smoking habits, prevalence of MetS, adherence to PA. guidelines, and fibre intake. Estimates are interpreted as the association of the sarcopenia risk score (SRS) with 0.1 g/BW increase of plant protein to the detriment of animal protein, while keeping total protein intake constant.
